# The complete mitochondrial genome of *Anelytra* (*Anelytra*) *eunigrifrons* (Orthoptera, Tettigoniidae: Conocephalinae)

**DOI:** 10.1128/mra.01414-25

**Published:** 2026-04-27

**Authors:** Guoqing Li, TingTing Yu, Xun Bian, Bin Zhang

**Affiliations:** 1 College of Life Sciences and Technology, Inner Mongolia Normal University 71203 , Hohhot, China; 2 Guangxi Key Laboratory of Rare and Endangered Animal Ecology, Guangxi Normal University 12388, Guilin, China; 3 Key Laboratory of Biodiversity Conservation and Sustainable Utilization for College and University of Inner Mongolia Autonomous Region, Hohhot, China; University of California Riverside 8790, Riverside, California, USA

**Keywords:** mitogenome, *Anelytra *(*Anelytra*)* eunigrifrons*, Conocephalinae

## Abstract

We report the complete mitochondrial genome of the insect *Anelytra* (*Anelytra*) *eunigrifrons* Ingrisch, 1998, based on a specimen from Menglai, Xima, Yunnan, China. The 15,492 bp AT-rich (66.6%) mitochondrial genome contains 13 protein-coding, 22 tRNA, and 2 rRNA genes, showing the same gene content as *Anelytra* (*Anelytra*) *obtusa*.

## ANNOUNCEMENT

Conocephalinae (Orthoptera: Tettigoniidae) is an insect subfamily with a cosmopolitan distribution from temperate to tropical regions worldwide (1, 2), while *Anelytra* species are mainly distributed in tropical and subtropical Asia (3). The genus is characterized by markedly shortened forewings and reduced hindwings (4). *Anelytra* (*Anelytra*) *eunigrifrons* was described by Ingrisch in 1998 from Myanmar (Shan State) in the Indochinese region (5), later recorded from India (Umiam, Meghalaya) in 2015 (6) and Yunnan, China, in 2021 (7), expanding its known range. To enrich molecular data and clarify the phylogeny of *Anelytra*, the complete mitochondrial genome of *Anelytra* (*Anelytra*) *eunigrifrons* was assembled and annotated.

The specimen of *Anelytra* (*Anelytra*) *eunigrifrons* was collected in Menglai, Xima, Yunnan, China (24.7628 N, 97.7438 W, 1,847 m), and the voucher is deposited at Guangxi Normal University. The DNA was extracted from the hind femur using the TIANamp Genomic DNA Kit (TIANGEN). A 150 bp paired-end library was prepared using the MGIEasy Kit (MGI) and subsequently sequenced on an Illumina NovaSeq 6000 platform (Illumina Inc.). The raw data were processed using fastp v0.20.0 (8) by trimming adapters and primers and removing reads with phred quality <Q5 or >3 N bases. The COX1 gene sequence from the mitochondrial genome of a congeneric species, *Anelytra obtusa* (NC_065466), was used as the seed sequence for *de novo* assembly with NOVOPlasty 4.3.5 (9). A total of 19,664 reads assembled into a single circular contig (15,492 bp), with an N50 equal to the assembly length, 33.4% GC content, and 258× average coverage. The assembled mitogenome was subsequently validated by BLAST search(10) against the NCBI database, which identified *A. obtusa* as the closest match. The genome was then annotated using Geneious Prime 2025.0.2 (https://www.geneious.com), with *A. obtusa* as the reference. The boundaries of protein-coding genes, rRNAs, and tRNAs were identified by comparison with the complete mitochondrial genomes of two congeneric species obtained from GenBank, and the initiation and termination codons were verified according to the invertebrate mitochondrial genetic code (11, 12). Nucleotide identities were calculated using BLAST searches under default settings. The mitochondrial genome map was graphically constructed and visualized using the online version of OGDRAW (13).

**Fig 1:**
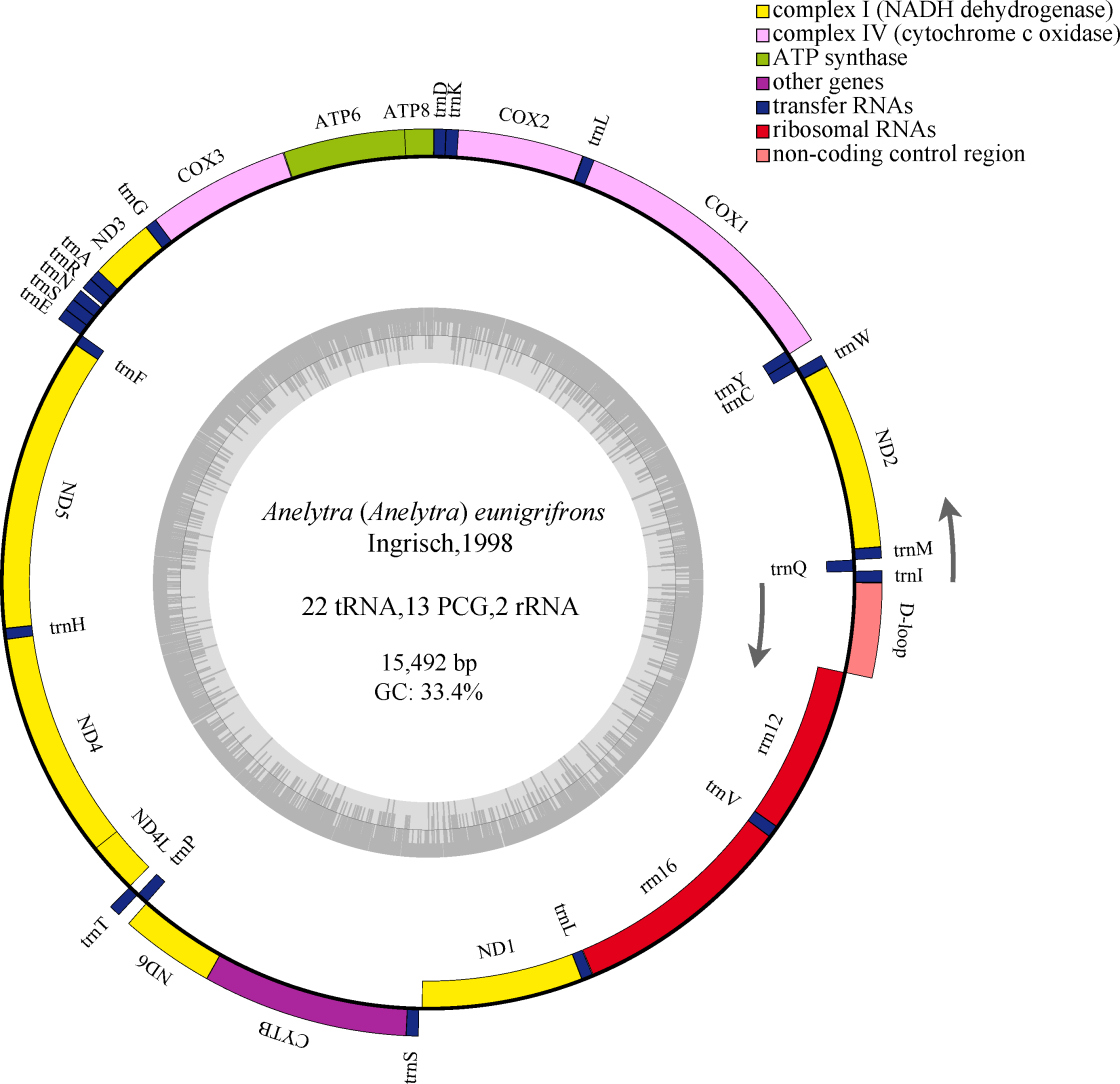
Circular map of the mitochondrial genome of *Anelytra* (*Anelytra*) *eunigrifrons* (5). The innermost ring shows the GC content in gray and the direction of transcription, as indicated by arrows. The outer ring represents genes, with clockwise-transcribed genes on the inside and counterclockwise-transcribed genes on the outside. Gene groups are color-coded as indicated in the key at the top right. PCG, protein-coding genes.

The complete circular mitochondrial genome of *Anelytra* (*Anelytra*) *eunigrifrons* is 15,492 bp in length and contains the typical 37 genes, including 13 protein-coding genes, 22 tRNA genes, and 2 rRNA genes, as well as a non-coding control region (D-loop) (Fig. 1). Six of the protein-coding genes initiate with ATG (ATP6, COX2, COX3, ND4, ND4L, and CYTB), three with ATT (ND2, ND3, and ND5) and ATC (COX1, ATP8, and ND6), and one with GTG (ND1) (Table 1). Four of the protein-coding genes terminate with TAA (ATP6, ND2, ND4L, and ND6), four with TAG (ATP8, ND1, ND3, and CYTB), two with TA (COX3 and ND4), and three genes (COX1, COX2, and ND5) with a single T. In orthopteran mitochondrial protein-coding genes, incomplete termination codons (T or TA) are usually completed through post-transcriptional polyadenylation, which restores a full TAA stop codon (14, 15).

**TABLE 1:** Gene composition and annotation of *Anelytra* (*Anelytra*) *eunigrifrons* (5)

Gene	Type	Minimum nucleotide position	Maximum nucleotide position	Length	Start codon	Stop Codon	Direction
tRNA-Ile	tRNA	1	66	66	–^*a*^	–	Forward
tRNA-Gln	tRNA	64	132	69	–	–	Reverse
tRNA-Met	tRNA	132	194	63	–	–	Forward
ND2	CDS	195	1226	1,032	ATT	TAA	Forward
tRNA-Trp	tRNA	1229	1294	66	–	–	Forward
tRNA-Cys	tRNA	1287	1352	66	–	–	Reverse
tRNA-Tyr	tRNA	1353	1417	65	–	–	Reverse
COX1	CDS	1392	2949	1,558	ATC	T	Forward
tRNA-Leu	tRNA	2950	3013	64	–	–	Forward
COX2	CDS	3016	3706	691	ATG	T	Forward
tRNA-Lys	tRNA	3707	3776	70	–	–	Forward
tRNA-Asp	tRNA	3776	3841	66	–	–	Forward
ATP8	CDS	3842	4003	162	ATC	TAG	Forward
ATP6	CDS	3997	4671	675	ATG	TAA	Forward
COX3	CDS	4675	5462	788	ATG	TA	Forward
tRNA-Gly	tRNA	5462	5526	65	–	–	Forward
ND3	CDS	5527	5880	354	ATT	TAG	Forward
tRNA-Ala	tRNA	5879	5940	62	–	–	Forward
tRNA-Arg	tRNA	5941	6003	63	–	–	Forward
tRNA-Asn	tRNA	6021	6085	65	–	–	Forward
tRNA-Ser	tRNA	6086	6152	67	–	–	Forward
tRNA-Glu	tRNA	6153	6218	66	–	–	Forward
tRNA-Phe	tRNA	6217	6280	64	–	–	Reverse
ND5	CDS	6281	8012	1,732	ATT	T	Reverse
tRNA-His	tRNA	8013	8076	64	–	–	Reverse
ND4	CDS	8076	9415	1,340	ATG	TA	Reverse
ND4L	CDS	9409	9705	297	ATG	TAA	Reverse
tRNA-Thr	tRNA	9708	9771	64	–	–	Forward
tRNA-Pro	tRNA	9771	9840	70	–	–	Reverse
ND6	CDS	9842	10363	522	ATC	TAA	Forward
CYTB	CDS	10363	11499	1,137	ATG	TAG	Forward
tRNA-Ser	tRNA	11498	11566	69	–	–	Forward
ND1	CDS	11583	12530	948	GTG	TAG	Reverse
tRNA-Leu	tRNA	12531	12596	66	–	–	Reverse
16S rRNA	rRNA	12600	13914	1,315	–	–	Reverse
tRNA-Val	tRNA	13915	13985	71	–	–	Reverse
12S rRNA	rRNA	13986	14967	982	–	–	Reverse
CR	D-LOOP	14968	15492	525	–	–	Forward

^a–^ , not applicable.

## Data Availability

The complete mitochondrial genome sequence of *Anelytra* (*Anelytra*) *eunigrifrons* is available in GenBank under accession number PX617820. The associated BioProject, BioSample, and SRA numbers are PRJNA1355332, SAMN53036655, and SRR35927703, respectively. The mitochondrial genome referenced in the text is *Anelytra* (*Anelytra*) *obtusa* with GenBank accession number NC_065466.
